# Bacteriophages of *Helicobacter pylori*

**DOI:** 10.3389/fmicb.2020.549084

**Published:** 2020-11-12

**Authors:** Angela B. Muñoz, Johanna Stepanian, Alba Alicia Trespalacios, Filipa F. Vale

**Affiliations:** ^1^Infectious Diseases Research Group, Microbiology Department, Sciences Faculty, Pontificia Universidad Javeriana, Bogotá, Colombia; ^2^Host–Pathogen Interactions Unit, Research Institute for Medicines (iMed-ULisboa), Faculdade de Farmácia, Universidade de Lisboa, Lisbon, Portugal

**Keywords:** *Helicobacter pylori*, bacteriophage, phage therapy, prophage genetic diversity, phage–host interaction

## Abstract

The bacterium *Helicobacter pylori* colonize the stomach in approximately half of the world’s population. Infection with this bacterium is associated with gastritis, peptic ulcer, adenocarcinoma, and gastric mucosa-associated lymphoid tissue lymphoma. Besides being a pathogen with worldwide prevalence, *H. pylori* show increasingly high antibiotic resistance rates, making the development of new therapeutic strategies against this bacterium challenging. Furthermore, *H. pylori* is a genetically diverse bacterium, which may be influenced by the presence of mobile genomic elements, including prophages. In this review, we analyze these issues and summarize various reports and findings related to phages and *H. pylori*, discussing the relationship between the presence of these elements and the genomic diversity, virulence, and fitness of this bacterium. We also analyze the state of the knowledge on the potential utility of bacteriophages as a therapeutic strategy for *H. pylori*.

## Introduction

*Helicobacter pylori* is a spiral-shaped, microaerophilic, Gram-negative, pathogenic bacterium that colonizes and persists in the human stomach. *H. pylori* infection is considered the most frequent chronic bacterial infection worldwide (Camilo et al., 2017). All individuals infected with *H. pylori* present with chronic gastritis, while 20% develop peptic ulcer and less than 1% develop adenocarcinoma or mucosa-associated lymphoid tissue (MALT)-type gastric lymphoma. Further, *H. pylori* infection is recognized as the leading risk factor for the development of gastric cancer (Polk and Peek, 2010). Numerous factors have been described as responsible for the progression of the infection to more severe diseases: although these factors may be inherent to the host (Figueiredo et al., 2017; Miftahussurur et al., 2017), several *H. pylori* virulence factors have also been associated with malignancy. The most frequently associated are the Cag pathogenicity island (*cagPAI*) and the VacA vacuolating cytotoxin (Denic et al., 2020).

*CagPAI* is a genetic locus of 40 kb, comprising 31 genes that encode a type IV secretion system (T4SS). The T4SS enables the injection of bacterial components, such as the CagA oncoprotein, into host gastric epithelial cells (Soluri et al., 2020). VacA is a toxin secreted by *H. pylori* that inserts into host cell membranes to form chloride-sensitive channels and disrupt endolysosomal trafficking, causing an accumulation of dysfunctional lysosomes and autophagosomes (Denic et al., 2020). Most *H. pylori* strains isolated have the *vacA* gene. However, differences in the alleles of the signal (s) and middle (m) regions of this gene are responsible for differing levels of cytotoxicity (Oktem-Okullu et al., 2020).

In addition to the virulence of *H. pylori*, another critical challenge for clinicians has been to find an optimal treatment that takes into account the high levels of resistance of *H. pylori* to antibiotics (Camargo et al., 2014; Talebi Bezmin Abadi, 2017). *H. pylori* cure rates using antibiotic treatment have been reported to be as low as 57% (Dos Santos and Carvalho, 2015). The minimum acceptable rate for a first attempt based treatment is 90% (Nijevitch et al., 2014; Jones et al., 2017). Current treatments are based on the use of antibiotics combined with a proton pump inhibitor (PPI) (Malfertheiner et al., 2017). The PPI is used to increase intragastric pH because, at an acidic pH, *H. pylori* transforms into its antibiotic-resistant coccoid form (Ierardi et al., 2019). Furthermore, acidic pH decreases the antimicrobial activity and half-life of antibiotics (Otero et al., 2018).

The increasing rates of *H. pylori* antibiotic resistance have necessitated the development of new therapeutic strategies (Vítor and Vale, 2011). An emerging alternative treatment for antibiotic-resistant bacterial infections is phage-therapy, which uses bacteriophages, also known as phages, to eliminate a bacterial population (Lin D. M. et al., 2017). The resurgence of this therapy has been due mainly to studies showing that phages are highly specific and easy to isolate (Loc-Carrillo and Abedon, 2011); moreover, phage therapy has demonstrated effectiveness in treating various infections, even curing chronic infections (Abedon, 2019). Despite the benefits of this therapy, its use for the treatment of *H. pylori* infection is a distant goal, considering that the understanding of *H. pylori* phage biology is still in its infancy.

Phages, which are viral particles that infect bacteria, may be lytic, lysogenic, or pseudo-lysogenic. Lytic phages recognize bacterial surfaces and inject their nucleic acids into the host cell; then, they assemble, multiply, and finally disrupt the cell to release phage progeny that infect new bacterial cells (Sulakvelidze, 2005). In lysogenic phages, phage DNA integrates into the bacterial genome, forming prophages (also known as template phages). Prophages promote bacterial evolution via horizontal gene transfer and induce the transduction of several genes involved in the biological behaviors of the bacterium (Brüssow et al., 2004; Paterson et al., 2010; Touchon et al., 2016). Pseudo-lysogenic phages are present as episomes (i.e., the genetic material is not integrated into the bacterial genome) and postpone cell lysis in nutrient-depleted hosts (Uchiyama et al., 2013); both lytic and lysogenic phages, under certain conditions, may acquire episomal forms (Łoś and Wȩgrzyn, 2012).

Bacteriophages represent the absolute majority of all organisms in the biosphere (Hatfull and Hendrix, 2011). Among the various bacteriophages, the discovery of *H. pylori* prophages has been of particular importance in explaining this bacterium’s tremendous genetic diversity. This review aims to collate and analyze relevant publications regarding phages related to *H. pylori* and to understand how these phages impact the diversity and virulence of this bacterium. Additionally, we summarize reports on lytic phages with activity against *H. pylori.*

### *H. pylori* Prophages

The first observations of phage-like intracellular particles in preparations of *H. pylori* were made shortly after the discovery of this bacterium (Marshall et al., 1987; Goodwin et al., 1989). In the early 1990s, a lysogenic strain of *H. pylori* that produced phage particles spontaneously was described (Schmid et al., 1990). Three years later, the lytic cycle of this phage was reproduced. Negative stain electron microscopy revealed that phage heads of around 50 to 60 nm and the DNA length was estimated to be 22,000 bp (Heintschel Von Heinegg et al., 1993).

With recent advances in sequencing technologies, studies aiming at the discovery of phages are increasing. Since 2011, investigations focusing on the analysis of *Helicobacter* spp. genomes have been published that corroborate the presence of prophages in the bacterial genome and analyze their functions in this context (Lehours et al., 2011; Luo et al., 2012; Uchiyama et al., 2012, 2013, 2016; Vale et al., 2015, 2017; Secka et al., 2017; Vale and Lehours, 2018) (Table 1). These studies report that prophages are present in around 20% of *H. pylori* isolates. The first of these studies reported a prophage sequence in *H. pylori* isolated from a patient with MALT lymphoma. This prophage, named PhiHp33, was isolated from *H. pylori* strain B45 and could be induced by UV light. Genomic sequence analysis demonstrated that *H. pylori* strains from different regions carry full or partial prophage sequences. Analysis of integrase and holin gene sequences allowed differentiation of strains according to their geographical origin, generating results consistent with classification based on multi-locus sequence typing (MLST) (Lehours et al., 2011; Vale et al., 2015; Secka et al., 2017).

**TABLE 1:** Main bacteriophages of *H. pylori.*

Bacteriophage	Type	Family	Genome size (Kb)	Details	References
1961P	Prophage	*Podoviridae*	26.8	Accession number: NC_019512.1 Head: 68–74 nm Tail: 23 × 13.3 nm	(Luo et al., 2012)
*Campylobacter piloridis* strain	Prophage	No data	No data	Intracellular particles of 40 nm in diameter	(Marshall et al., 1987)
*Campylobacter pylori* strain	Prophage	No data	No data	Intracellular particles of 85 nm in diameter	(Goodwin et al., 1989)
De-M53-M	Prophages	No data	28.1	Prophage population: hpNEurope Accession number: KX119205	(Vale et al., 2017)
Fr-ANT170-U	Prophage	No data	31.2	Prophage population: hpAfrica1 Accession number: KX119201	(Vale et al., 2017)
Fr-B41-M	Prophage	No data	29.4	Prophage population: hpSWEurope Accession number: KX119190	(Vale et al., 2017)
Fr-B58-M	Prophage	No data	22.6	Prophage population: hpEastAsia Accession number: KX119193	(Vale et al., 2017)
Fr-G12-G	Prophage	No data	28.6	Prophage population: hpEastAsia Accession number: KX119194	(Vale et al., 2017)
Fr-GC43-A	Prophage	No data	33.0	Prophage population: hpEastAsia Accession number: KX119195	(Vale et al., 2017)
Fr-MEG235-U	Prophage	No data	31.2	Prophage population: hpAfrica1 Accession number: KX119200	(Vale et al., 2017)
HP1	Prophage	*Siphoviridae*	22	Head:50–60 nm Tail:170 × 9.5 nm	(Heintschel Von Heinegg et al., 1993)
KHP30	Prophage	Cannot be classified / new family	26.2	Episome Accession number: NC_019928.1 Head: 67–71 nm Without tail	(Uchiyama et al., 2012, 2013; Takeuchi et al., 2018)
KHP40	Prophage	No data	26.4	KHP30-like Accession number: NC_019931.1	(Uchiyama et al., 2012)
PhiHp33	Prophage	*Siphoviridae*	24.6	Inducible by UV light Accession number: NC_016568 Head: 55–70 nm Tail: 92 × 6 nm	(Lehours et al., 2011)
Pt-1293-U	Prophage	No data	30.1	Prophage population: hpAfrica1 Accession number: KX119202	(Vale et al., 2017)
Pt-1846-U	Prophage	No data	28.0	Prophage population: hpAfrica1 Accession number: KX119176	(Vale et al., 2017)
Pt-1918-U	Prophage	No data	28.7	Prophage population: hpSWEurope Accession number: KX119192	(Vale et al., 2017)
Pt-212-99R-U	Prophage	No data	23.0	Prophage population: hpAfrica1 Accession number: KX119193	(Vale et al., 2017)
Pt-228_99-G	Prophage	No data	30.1	Prophage population: hpAfrica1 Accession number: KX119175	(Vale et al., 2017)
Pt-4472-G	Prophage	No data	27.6	Prophage population: hpSWEurope Accession number: KX119190	(Vale et al., 2017)
Pt-4481-G	Prophage	No data	25.4	Prophage population: hpAfrica1 Accession number: KX119196	(Vale et al., 2017)
Pt-4497-U	Prophage	No data	29.4	Prophage population: hpSWEurope Accession number: KX119191	(Vale et al., 2017)
Pt-5322-G	Prophage	No data	28.3	Prophage population: hpAfrica1 Accession number: KX119198	(Vale et al., 2017)
Pt-5771-G	Prophage	No data	29.8	Prophage population: hpAfrica1 Accession number: KX119199	(Vale et al., 2017)
Pt-B89-G	Prophage	No data	27.4	Prophage population: hpAfrica1 Accession number: KX119203	(Vale et al., 2017)
Pt-B92-G	Prophage	No data	30.5	Prophage population: hpAfrica1 Accession number: KX119197	(Vale et al., 2017)
Sw-A626-G	Prophage	No data	31	Prophage population: hpNEurope Accession number: KX119177	(Vale et al., 2017)
Sw-577-G	Prophage	No data	26.9	Prophage population: hpNEurope Accession number: KX119204	(Vale et al., 2017)
UK-EN31-U	Prophage	No data	30.5	Prophage population: hpNEurope Accession number: KX119174	(Vale et al., 2017)
UK-EN32-U	Prophages	No data	29.9	Prophage population: hpNEurope Accession number: KX119206	(Vale et al., 2017)
ϕ HPE1	Lytic	*Podoviridae*	No data	Titer: 10^9^ PFU/ml Head: 62 nm Short noncontractile tail: 12 × 6 nm	(Abdel-Haliem and Askora, 2013)
ϕ HPE2	Lytic	*Siphoviridae*	No data	Titer: 10^10^ PFU/ml Head: 95 nm Tail: 180 × 15 nm	(Abdel-Haliem and Askora, 2013)
Hp φ	Lytic	No data	No data	Used in synergy with lactoferricin and hydroxyapatite. Adsorption rate: 1.89 × 109 mL/min Latent period: 45 min Burst size: 80 PFU	(Cuomo et al., 2020)
*H. pylori* strain BAPOUI	Lytic	No data	No data	Obtained from human feces Head: 100 nm Without tail	(Vale et al., 2008)

In 2012, the isolation of a new phage (1961P) from Taiwanese *H. pylori* strains was reported. This phage showed characteristics compatible with belonging to the family *Podoviridae*. Further, sequence analysis identified other similar prophages integrated into the genomes of different *H. pylori* strains that had previously been sequenced (Luo et al., 2012). Additionally, in the same year, the complete genome sequences of two *H. pylori* bacteriophages (KHP30 and KHP40) isolated from Japanese patients were reported. These prophages were obtained after spontaneous release of viral particles by *H. pylori* strains (Uchiyama et al., 2012). The KHP30 phage was characterized as spherical, with a lipid envelope. It was present in infected bacteria as an episome, indicating that it likely represents a new phage family (Uchiyama et al., 2013). Subsequently, the possibility of lysogeny of active KHP30-like phages in 174 Japanese *H. pylori* strains was examined because their genomes contain a putative integrase gene; one strain, NY40, was found to include a KHP30-like prophage sequence. In this strain, the attachment sites, *attL* and *attR*, were almost the same place in the genome. Further, their sequences were detected previously by Lehours et al., suggesting an active parental KHP30-like phage integrated into the ancestral NY40 genome in a site-specific manner (Uchiyama et al., 2016).

Analysis of KHP30 has continued, with researchers investigating its effects on the behaviors of the host strain (NY43) and demonstrating differences between the characteristics of *H. pylori* strains infected with prophage and novel prophage-free derivative strains. A recent study indicated that the presence of bacteriophages induces genetic alterations in the host genome, leading to the continuous production of prophage-free derivatives that coexist in *H. pylori* microbial communities and confer diversity in morphology, motility, viability, and pathogenicity. Further, this study reported that KHP30 prophage could specifically suppress CagA expression (Takeuchi et al., 2018). Another study conducted using an *H. pylori* strain isolated from an ethnic minority patient in China revealed the presence of a 32.5 kb prophage integrated into the genome and demonstrated that most of its genes (30/33) were the same as those of KHP30; however, in this case, the prophage was inserted between two putative virulence genes, *oipA* and *homB*, which encode adherence factors that mediate interactions between *H. pylori* and the host microenvironment. The authors suggest that the presence of a prophage inserted in this region indicates that HomB and OipA may act as receptors for the phage (You et al., 2015).

In addition to the characterization of *H. pylori* prophages, the relationship between the presence of prophages and bacterial genetic diversity has been widely discussed. *H. pylori* is a genetically diverse species. This diversity has been associated with the geographic origin of populations (Matta et al., 2017) and likely influenced by the presence of mobile genomic elements, such as prophages, among other factors (Vale and Lehours, 2018). Since 2015, Vale et al. have reported that *H. pylori* prophages may contribute to bacterial genetic diversity. Most observations indicated a phylogeographic agreement between phage and bacterial genes, suggesting a co-evolution model between the virus and its bacterial host. Nonetheless, some prophages were assigned to populations distinct from their host, while others exhibited recombination signs among populations (Yahara et al., 2019). The phylogeographic distribution of *H. pylori* prophages can be characterized by Bayesian clustering analysis, based on a typing scheme that includes the sequences of two prophage genes: integrase (which is responsible for the integration of the phage genome into the bacterial chromosome) and holin (which is involved in cell lysis during a lytic cycle). Four prophage populations have been described: hpAfrica1, hpEastAsia, hpNEurope, and hpSWEurope, notably subdividing the European population (Vale et al., 2015). Recently, based on the whole-genome analysis of *H. pylori*, two subpopulations of hpEurope were also observed (Thorell et al., 2017).

Available information on *H. pylori* prophage diversity indicates that prophages and bacteria share a complex evolutionary history (Vale et al., 2017) and that the bacterial genome has been widely modified, in diverse regions, via horizontal gene transfer (Takeuchi et al., 2018). A few studies have investigated the presence of phages in other *H. pylori* strains; for example, a study from 2016 reported the detection of a 31.7 kb prophage in an *H. pylori* strain isolated from a Mexican patient with gastric cancer (Mucito-Varela et al., 2016). Further, Kumar et al. (2017) analyzed *H. pylori* strains from Kuwait and reported a prophage in a strain isolated from a patient with active chronic gastritis and focal intestinal metaplasia.

In silico analyses have revealed prophage orthologous genes in the genomes of bacterial strains with virulence genes such as *cagA* or *vacA* (Kyrillos et al., 2016). Reports suggest that prophages inserted in the *H. pylori* genome represent essential elements for the adaptation of this bacterium to hostile environments, because metabolic advantages or tolerance mechanisms that can be influenced by prophages may improve *H. pylori* competitiveness. Nevertheless, no specific prophage functions have, as yet, been recognized in *H. pylori*, and the roles of prophage in disease have rarely been recorded (Vale et al., 2017). Long-term colonization of the human stomach by *H. pylori* has allowed it to evolve together with its human host for more than 100,000 years (Moodley et al., 2012). Furthermore, because of frequent mutation and recombination events and horizontal gene transfer events, *H. pylori* is an extremely diverse bacterium. Specifically, *H. pylori* is naturally competent for transformation and highly competent in recombination, making the exchange of chromosomal DNA fragments between strains frequent and highly efficient (Fischer et al., 2020). Phages have generally been considered as actively involved in the processes of recombination and horizontal genetic exchange. In this context, a recent study presented a quantitative analysis of the described recombination events of *H. pylori* phage genes, revealing frequent recombination among phage core genes previously reported as less prone to recombination. This observation appears to reflect the co-evolutionary relationships of these phages with their hosts, revealing the evolutionary arms race that exists, either to contribute to phage escape from bacterial immunity or to protect hosts that produce defective phages. The high recombination rate in *H. pylori* phages suggests that they are among the most recombinogenic phages on earth (Yahara et al., 2019). To further advance knowledge of this topic, it is important to investigate the biological and genomic characteristics of *H. pylori* phages in detail. Likewise, valuable information may emerge from the continued search for phage in strains associated with different pathologies and from various geographical locations. Furthermore, important insights may be obtained through investigation of phage insertion sites and their roles in the bacterial genome, considering that prophages can act as gene reservoirs, which may benefit pathogens in ways that are just beginning to be determined.

### Lytic Bacteriophages of *H. pylori*

The rise of antibiotic resistance increased the interest in studying bacteriophages, particularly the lytic ones. Indeed, phage therapy has various potential advantages than antibiotic use because phages and phage lysins, are highly specific, affecting the target strain but not the microbiome (Brüssow, 2017). Further, phages only replicate at the infection site, and no secondary effects have been described (Matsuzaki et al., 2005). Although these findings indicate the potential to use phages to eradicate *H. pylori*, reports in this field are scarce.

At the time of this review, there were only three studies on lytic bacteriophages (Table 1): the first was published by Vale et al. (2008), who isolated one lytic phage from human feces. Not many characteristics of this phage are known. It was only reported that it is a phage without a tail and an approximate size of 100 nm. The second study was published in 2013 by Abdel-Haliem and Askora, who isolated and characterized two lytic phages (ΦHPE1 and ΦHPE2) from wastewater (Abdel-Haliem and Askora, 2013). These phages were classified, by electronic microscopy, as belonging to the *Podoviridae* and *Siphoviridae* families, respectively. The titers for ΦHPE1 and ΦHPE2 were 10^9^ PFU/ml and 10^10^ PFU/ml, respectively. These studies did not offer data on the latent period, burst size, or antimicrobial potential of these phages.

A third recent study by Cuomo et al. (2020) describes a new therapeutic option based on using a lytic phage of *H. pylori* (Hp φ), isolated from gastric biopsies. The phage was tested alone and combined with lactoferrin (LA) and adsorbed on hydroxyapatite (HA) nanoparticles. The characterization of Hp φ revealed that the adsorption rate was 1.89 × 10^9^ mL/min, the latent period was 45 min, and the burst size was only 80 PFU. These analyses showed that the use of the complex (Hp φ + LF-HA) enhances the activity of Hp φ up to 4 times. The use of HA as a vehicle can improve the natural properties of both Hp φ and LF because it protects the gastric acid environment. An important finding, considering that phages are often sensitive to extreme pH values such as those associated with stomach acidity (Da̧browska and Abedon, 2019).

The studies available to date have not addressed certain aspects important for defining phage- therapy. The genome sequences of the phages remain unavailable. Genomic analysis could determine whether the phage genome comprises genes involved in lysogenic events, encode toxins, or represent determinants of antimicrobial resistance, or whether they code for lytic cycle repressors, or site-specific integrases or recombinases (Fernández et al., 2019). Likewise, considering the specific defense mechanisms mounted by bacteria against bacteriophage infections, it is essential to address the possible resistance that *H. pylori* may develop against these phages (Hyman and Abedon, 2010). The study by Cuomo et al. (2020) does not define the minimum effective combined doses of Hp φ and LF-HA.

Additional to the use of lytic phages, the use of phage lysins and related bacteriolytic enzymes are also being considered in phage therapy (Fischetti, 2018). Many phages have polysaccharide depolymerases and lysins that recognize, bind, and degrade the polysaccharide compounds (Latka et al., 2017). The enzyme holin could also be used as a therapeutic alternative. This enzyme is involved in the holin-endolysin pathway responsible for the first steps of bacterial lysis, which begins when the holin forms micrometer-scale holes in the bacterial inner membrane, releasing active endolysin into the periplasm to degrade peptidoglycan (Cahill and Young, 2019). Studies have evaluated the effect of enzymes for the treatment of bacterial infections mainly caused by gram-negative bacteria, and have shown efficacy in reducing bacterial virulence (D’andrea et al., 2017; Lin H. et al., 2017; Majkowska-Skrobek et al., 2018; Solovieva et al., 2018). Considering the presence of prophages in *H. pylori* and the background in other bacterial models, the investigation of these kinds of enzymes in *H. pylori* is feasible.

## Conclusion

The findings reviewed here show that *H. pylori* genetic diversity is influenced by prophages, which are frequently found in strains of this bacterium, represent a structured population, and are highly recombinogenic. The additional genetic diversity that phages may provide to *H. pylori* confers advantages to this bacterium in terms of persistence and colonization of different human populations. The study of *H. pylori* phages has gained importance considering the substantial challenges posed by infection with this bacterium, including low eradication rates and antibiotic resistance. Phages appear to be a promising approach for the design of future therapies. Advances in research to improve understanding of phage and *H. pylori* interactions are required, and studies investigating whether prophage genes belonging to the lysis cassette are useful for phage-therapy are warranted. Further, identification of more lytic phages of *H. pylori* would allow considering the phage-therapy as an alternative approach for eradicating *H. pylori*, which would undoubtedly mark a milestone in the history of applications of *H. pylori* phages.

## Author Contributions

AM, JS, AT, and FV conceived the review. AM and JS collected the information. AM and FV wrote the manuscript. All authors contributed to the manuscript revision, read, and approved the submitted version.

## Conflict of Interest

The authors declare that the research was conducted in the absence of any commercial or financial relationships that could be construed as a potential conflict of interest.
